# 2-[^18^F]-FDG PET/CT in rasmussen encephalitis: guiding diagnosis and treatment in a young adult with sudden onset

**DOI:** 10.22038/aojnmb.2025.84963.1606

**Published:** 2025

**Authors:** Mattoli Maria Vittoria, Francesca Serani, Stefano L. Sensi

**Affiliations:** 1 Nuclear Medicine Unit, Presidio Ospedaliero Santo Spirito, Pescara, Italy; 2 Department of Medicine (DIMED), Nuclear Medicine Unit, University of Padova, Padova, Italy; 3 Department of Neuroscience, Imaging and Clinical Sciences, University G. d'Annunzio of Chieti-Pescara, Chieti, Italy; 4 Epilespy Center, “SS Annunziata” Hospital of Chieti, University “G. d'Annunzio” of Chieti-Pescara, Chieti, Italy

**Keywords:** Neuroimaging, Cerebral FDG PET Autoimmune Encephalitis

## Abstract

Rasmussen encephalitis (RE) is a rare condition characterized by a chronic inflammatory disorder due to unilateral inflammation of the cerebral cortex. Typically, it involves one cerebral hemisphere and manifests through intractable epileptic seizures. Its occurrence in adults is infrequent. We present a case of a 28-year-old woman who was brought to the emergency room due to the sudden onset of uncontrolled seizures. The use of 2-[^18^F]-FDG PET/CT (FDG-PET) helped in the differential diagnosis between autoimmune seronegative encephalitis and Rasmussen encephalitis. Even though FDG-PET is not a mandatory diagnostic criterion in the clinical evaluation of RE patients, the presented case and the available literature suggest its usefulness as a valuable diagnostic tool in patients with uncertain diagnosis, emphasizing its potential as a reliable adjunct in challenging diagnostic scenarios and for patient follow-up.

## Introduction

Rasmussen encephalitis is a rare condition characterized by a chronic inflammatory disorder due to unilateral inflammation of the cerebral cortex. Typically, it involves one cerebral hemisphere and manifests through intractable epileptic seizures. The progressive course of the inflammation is mainly due to an immune-mediated process, even though no serological or intrathecal markers have been identified. Pharmacoresistant epilepsy, epilepsia partialis continua (EPC), progressive cognitive impairment, and hemiplegia are the hallmarks of RE. These symptoms evolve progressively: a prodromal stage with mild hemiparesis or infrequent seizures, an acute stage with frequent focal aware seizures and EPC, and a final stage with severe motor and cognitive deficits along with pharmacoresistant epilepsy (1).

 According to the 2005 European Consensus (2), three cardinal criteria are required for RE diagnosis: (1) focal seizures with unilateral cortical deficits, (2) unilateral EEG abnormalities, and (3) mono-hemispheric MRI focal cortical atrophy with grey and/or white matter hyperintense signals or atrophy of the ipsilateral head of the caudate nucleus. Alternatively, a diagnosis may be made if two of the following criteria are met: (1) EPC or progressive unilateral cortical deficits, (2) progressive mono-hemispheric cortical atrophy, or (3) histopathological features of RE on brain biopsy.

 Patients with late-onset Rasmussen encephalitis (lo-RE) may exhibit atypical clinical (e.g., slower evolution and delayed deficits) and neuroradiological (e.g., delayed focal cortical atrophy) features, complicating the diagnostic process (3-5). Up to 30% of lo-RE patients do not fulfill the classical diagnostic criteria. Although MRI is generally sufficient to support RE diagnosis in childhood-onset cases, its specificity is questionable in atypical presentations such as lo-RE. In these scenarios, FDG-PET-despite not being included as a mandatory criterion-is valuable, particularly when MRI findings are inconclusive. Previous reports have emphasized the potential role of FDG-PET in RE diagnosis (6-8). Moreover, FDG-PET/CT may also help differentiate RE from other conditions, such as autoimmune seronegative encephalitis, which has been shown to exhibit distinct metabolic patterns on FDG-PET (17-19). In this paper, we describe a case of lo-RE in which FDG-PET was critical in guiding treatment and confirming the diagnosis.

## Case Presentation

 A 28-year-old female with a past medical history of mild anxiety was brought to the emergency room due to the sudden onset of repetitive, unremitting clonic movements involving the right ocular orbicular muscle and the right upper arm. An EEG revealed bursts of high amplitude 5 Hz theta sequences with superimposed diphasic sharp waves over the bilateral frontotemporal derivations, leading to a diagnosis of focal motor status epilepticus. Intravenous boluses of lorazepam and lacosamide were initiated, resulting in temporary seizure control; however, seizures recurred over subsequent days with increased frequency and duration.

 Brain MRI demonstrated mild cortical atrophy predominantly affecting the left frontal and insular cortices. Cerebrospinal fluid analysis showed lymphocytic leukocytosis, an increased IgG ratio, and a negative polymerase chain reaction for neurotropic viruses (herpes simplex virus 1–2, herpes zoster, Epstein-Barr virus, cytomegalovirus). Extensive autoantibody panels performed on serum and CSF were unremarkable, and neuro-psychological evaluation revealed moderate attention deficits with impaired executive functions.

**Figure 1:**
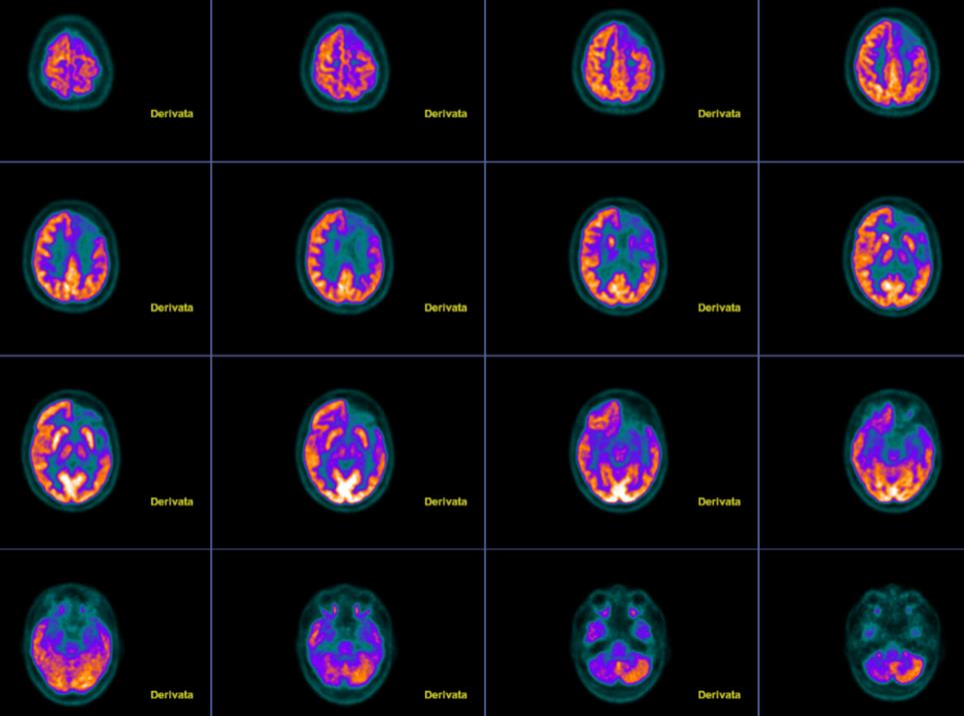
FDG-PET images with transaxial image: from the left upper corner in the upper row a diffuse unilateral hypometabolism in the left frontal lobe is showed. This pattern becomes more evident in the second row where the hypometabolism in the insular lobe and moderate hypometabolism in the left temporal lobe can be seen. At the bottom right corner, crossed cerebellar diaschisis is evident

 To differentiate between autoimmune seronegative encephalitis and Rasmussen encephalitis, a brain FDG-PET scan was performed. The patient fasted for 6 hours and underwent FDG-PET (acquisition time: 15 minutes) 30 minutes after injection of 185 MBq of tracer. PET images were qualitatively and semi-quantitatively assessed using automated 3D-SSP software CortexID Suite (GE Healthcare, Chicago, IL, USA), with Z-scores calculated from its normative database of 294 healthy, containing also age-matched controls (considered significant when < -2.0 SD). FDG-PET revealed diffuse unilateral hypometabolism, marked in the left frontal and insular lobes and moderate in the left temporal lobe, along with associated cortical atrophy, enlarged loco-regional CSF spaces, slight hypometabolism in the left caudate nucleus and thalamus, and a decrease in contralateral cerebellar metabolism (crossed cerebellar diaschisis) (Figure 1). CT images additionally showed an enlarged frontal sinus. This pattern, confirmed by semi-quantitative analysis with CortexID Suite, was suggestive of Rasmussen encephalitis.

 The patient was treated with intravenous immunoglobulins (0.4 g/kg per 3 days), resulting in complete seizure control. At 6-month follow-up, the patient experienced a mild recurrence of focal motor seizures (1-2 episodes/week) affecting the right ocular orbicular muscle and right upper arm. A new cycle of intravenous immunoglobulins produced positive clinical outcomes. Although a left functional hemispherectomy was proposed, the patient declined surgery. Consequently, a conservative approach with immunotherapy (azathioprine 100 mg/die) was initiated, leading to discrete control of neurological symptoms (approximately 1 seizure episode per month).

## Discussion

 FDG-PET is a useful diagnostic tool when there is clinical suspicion of Rasmussen encephalitis or when therapeutic decisions are uncertain. Although FDG-PET is not a mandatory diagnostic criterion per the 2005 European Consensus (2), it is recommended when confirming the uni-hemispheric nature of RE findings on structural imaging is needed. In fact, Villeneuve et al. (9) confirmed this recommendation in the 2022 revised diagnostic criteria for RE.

 RE is a rare neuroinflammatory condition with diverse neurological manifestations that complicate diagnosis and treatment (10). Immunomodulatory agents, such as steroids and immunoglobulins, are generally employed as first-line therapy, while neurosurgical interventions (e.g., hemispherectomy) are considered in severe cases-especially those with EPC. Early surgical treatment may improve cognitive outcomes, but it may also lead to subsequent IQ decline (10).

 There is limited research on noninvasive diagnostic methods for RE, particularly FDG-PET, with most data derived from case reports and small retrospective studies supporting its role (6, 11-13). For instance, Lee et al. (14) demonstrated that FDG-PET could detect focal metabolic abnormalities in pediatric patients with histologically confirmed RE and unremarkable MRI findings. The study highlighted differences in metabolic patterns between early (≤1 year) and late stages (>1 year) of the disease. Similarly, Vivek et al. (8) showed that simultaneous FDG-PET and MRI acquisition can help guide biopsy targets in patients with subtle structural findings. Fiorella et al. (7) reported that FDG-PET increases diagnostic confidence by unequivocally identifying the affected cerebral hemisphere in cases with ambiguous MRI findings. A multicenter study by Fogarasi et al. (15) further demonstrated that FDG-PET detects metabolic alterations earlier and more extensively than MRI. Guan et al. (10) and Wang et al. (16) emphasized the importance of FDG-PET (along with magnetoencephalography) in planning hemispherectomy by confirming the affected hemisphere through altered metabolism.

 Differentiating RE from autoimmune seronegative encephalitis is challenging, as both conditions may present with hypometabolic patterns on FDG-PET. However, autoimmune encephalitis typically shows bilateral or diffuse abnormalities with prominent behavioral changes and subacute encephalopathy, whereas RE exhibits a unilateral pattern correlating with focal deficits and cortical atrophy. In our patient, the unilateral hypometabolism and mild cortical atrophy strongly supported the diagnosis of RE. Recent studies specifically addressing FDG-PET patterns in autoimmune encephalitis (17–19) further underscore these differences. Comprehensive clinical evaluation-including EEG, detailed history, and objective imaging analysis-is therefore essential for accurate diagnosis.

## Conclusion

 Although FDG-PET is not a mandatory diagnostic criterion for RE, this case and the available literature demonstrate its value as a diagnostic and management tool in patients with uncertain findings. FDG-PET confirmed the treatment decision in our patient by identifying a unilateral hypometabolic pattern consistent with RE and provided crucial information when MRI findings were inconclusive. Additionally, FDG-PET is valuable for follow-up and guiding therapeutic decisions, such as choosing between hemispherectomy and ongoing immunosuppressive therapy. Future multi-center, prospective studies are needed to further validate the role of FDG-PET in RE.
